# Parachordoma of Soft Tissues of the Arm: A Very Rare Tumour

**DOI:** 10.1155/2013/252376

**Published:** 2013-09-03

**Authors:** Vicente Estrems Díaz, Francesc Xavier Bertó Martí, Víctor Zarzuela Sánchez, Maria Isabel Cabanes Ferrer, Antonio Bru Pomer

**Affiliations:** Orthopaedic Department, Consorcio Hospital General Universitario de Valencia, Avenida Tres Cruces 2, 46014 Valencia, Spain

## Abstract

Parachordoma is an infrequent neoplasm that bears some histologic resemblance to chordoma. It affects both sexes, occurs typically during the fourth decade of life, and tends to present as a slow-growing painless mass at the level of the soft tissues of the extremities. Diagnosis should be based on immunohistochemical and cytogenetic studies, as the findings of imaging techniques are often unspecific. Although it is considered a benign lesion, its behavior tends to be locally aggressive, with reports of a recurrence rate of up to 20% and of several cases of metastasis. Fewer than 60 cases have been published in the English-speaking literature. In this paper we present the case of a 32-year-old male with a two-year history of parachordoma in the right wrist.

## 1. Introduction

Parachordoma is an infrequent benign neoplasm of uncertain origin. It is morphologically reminiscent of chordoma, but its location is extra axial. It affects both males and females of different ages and usually presents as a slow-growing mass at the level of the soft tissues of the extremities.

Fewer than 60 cases have been published in the English-speaking literature. The purpose of this study is to present a new case of parachordoma, laying special emphasis on the importance of a judicious differential diagnosis. A review of the literature on the subject is also presented.

## 2. Case Presentation

The patient was a 32-year-old male with no relevant medical history who presented to our department in December 2009 with a two-year history of a growing neoplasm in the dorsum of the wrist. The lesion had developed slowly and was not related to a traumatic event. The patient did not mention any recent episodes of fever, weakness of weight loss. 

Our initial examination revealed a firm neoplasm on the dorsal radial metaphysis fixed to the deep planes but not to the skin. There was thus no skin involvement or local redness. Pain was elicited on palpation and on finger flexion, but no pain was present at rest. 

**Figure 1:**
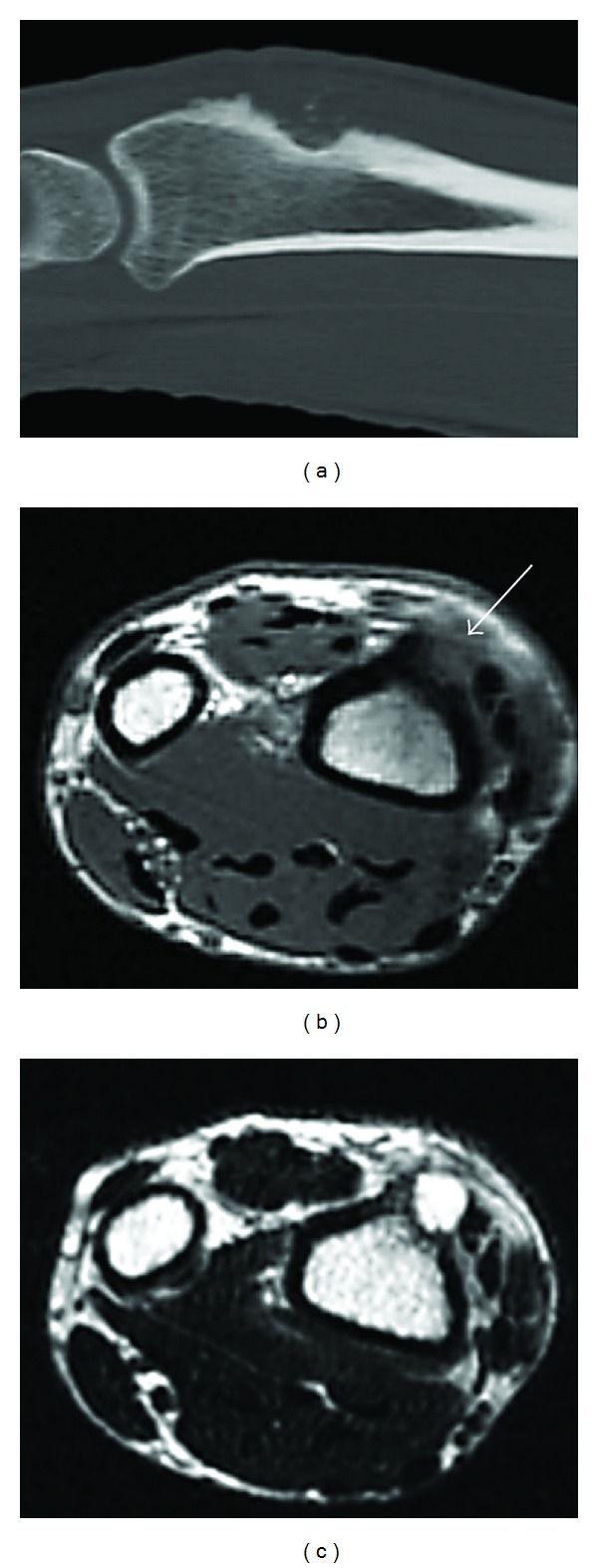
Imaging studies. (a) Sagittal CT-scan showing a lytic lesion on the dorsal cortex of the distal radius containing punctiform calcifications. ((b)-(c)) Axial MR images showing a soft tissue component (arrow) associated with the cortical lesion. Its appearance is isointense on T1 (b) and hyperintense on T2-weighted images (c).

The patient submitted an earlier computed tomography (CT) scan that showed a lytic lesion of 1.5 cm in diameter and well-defined margins affecting the dorsal cortex of the distal radius (Figure 1(a)). The imaging analysis was completed by a magnetic resonance imaging (MR) exam that revealed a soft tissue neoplasm, hyperintense on T2-weighted and STIR sequences, and isointense on T1-weighted sequences, located deep in the extensor compartment that had destroyed the dorsal radial cortex with no bone marrow involvement (Figures 1(b) and 1(c)). All analytic values were within the normal range.

In the face of these findings, the differential diagnosis included a wide variety of entities including benign conditions ranging from a fibrous cortical defect or a mixed tumor with a stromal component (myoepithelioma) to a soft tissue sarcoma or an osseous sarcoma with an associated soft tissue component (chondrosarcoma, osteosarcoma, and metastasis).

**Figure 2:**
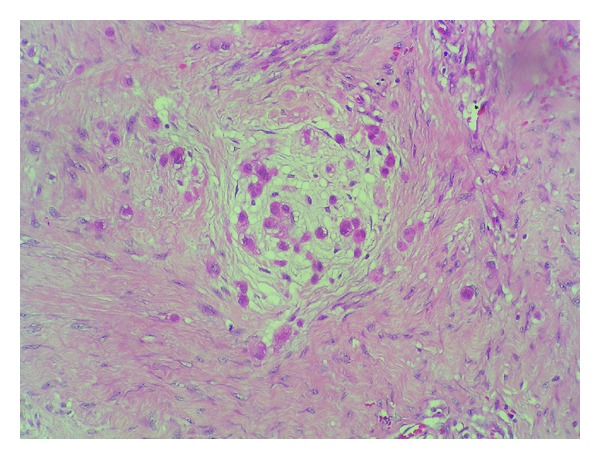
Histopathologic image showing how cells are arranged in a series of nidi, separated by abundant collagen bands.

An ultrasound-guided percutaneous biopsy was performed, which revealed a neoplastic proliferation comprising nidi and cords of eosinophilic cytoplasmic cells separated by dense fibrous stroma (Figure 2). In other areas, the cells displayed a spindled morphology. On the immunohistochemical study, the cells showed a positive reaction to CAM 5.2, protein S-100, cytokeratins, and the epithelial membrane antigen (EMA) and a negative reaction to CD31, CD34, actin, desmin, MYOD1, and HMB-45. These findings resulted in a final diagnosis of parachordoma. 

Prior to surgery, a thoracic-abdominal-pelvic CT scan was conducted to rule out the presence of additional lesions. 

**Figure 3:**
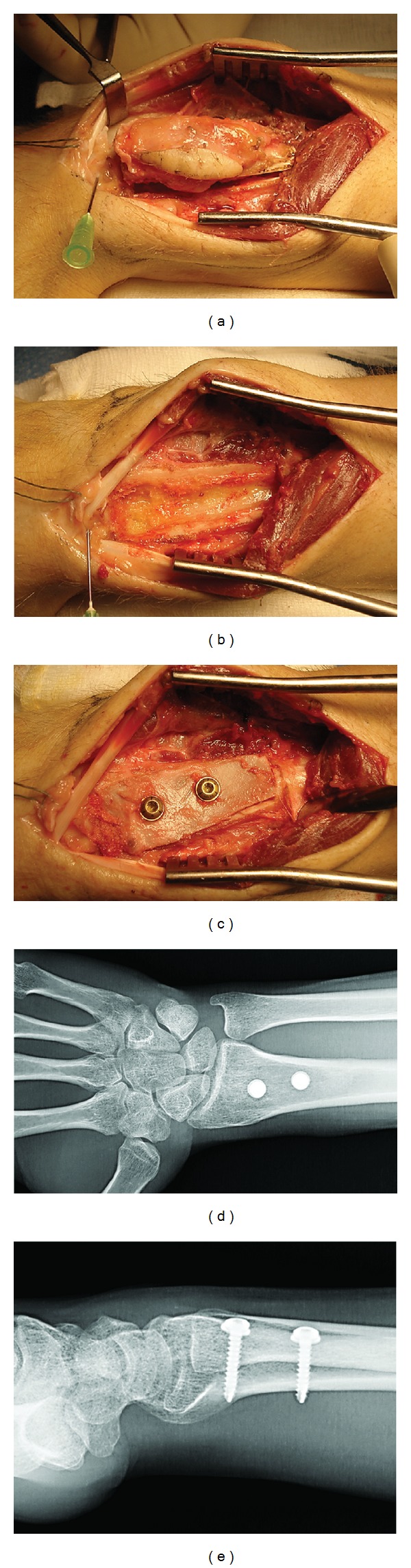
((a)–(c)) Intraoperative images. (a) En bloc resection of the biopsied area, soft tissue neoplasm, and osseous lesion. (b) Transverse semicircular osteotomy and curettage of the distal radial metaphysis. (c) Iliac crest allograft stabilized with two 3.5 mm. screws. ((d)-(e)) AP and lateral radiographs of the wrist three years after operation showing complete graft incorporation.

During the operation, an en bloc resection of the soft tissue neoplasm was performed as well as an excision of the associated bone lesion, which required a semicircular transverse osteotomy on the dorsal aspect of the distal radius. The bone defect was filled with a strut allograft from the iliac crest that was shaped to measure and stabilized with two 3.5 mm screws (Figures 3(a)–3(c)). The histologic study of the resected specimen confirmed the diagnosis of parachordoma as well as the presence of negative resection margins.

Three years after the procedure, the patient is asymptomatic with wrist range of motion of 110° for flexion-extension, 160° for pronosupination, a 60° radial/ulnar tilt arc; the pain score is 0 on the visual analog scale. Radiographs show complete graft incorporation (Figures 3(d) and 3(e)) with no signs that the condition has recurred.

## 3. Discussion

Parachordoma is an extremely rare soft tissue tumor first reported by Laskowski in 1955 and revisited by Dabska in 1977 [1]. Fewer than 60 cases have been documented to date in the English-speaking literature. The condition affects both sexes, although it is slightly more common in males than in females. Mean age at presentation is 34.4 years (range: 4–86) [2].

The most common location of parachordoma is the soft tissues of the extremities (78%), followed by the chest, the trunk, and the pelvis [2]. Clinically, it presents as a painless slow-growing nodular mass, which means that it usually goes unnoticed, and so diagnosis is normally established at a late stage.

The CT study shows a well-defined homogeneous, or slightly heterogeneous soft tissue neoplasm that may present with calcifications. The destruction of adjacent osseous structures seen in our case is highly unusual; only two similar cases have been reported in the literature [3, 4]. On MRi, the lesion appears as hypo- or isointense on T1-weighted sequences and as hyperintense on T2 and STIR images [2–4]; it can sometimes be surrounded by edematous soft tissues [2].

The most crucial factor about parachordoma is its differential diagnosis. Conditions such as extraskeletal myxoid chondrosarcoma, epithelioid sarcoma, and metastatic clear cell carcinoma must be ruled out. Nevertheless, the main controversy lies in whether parachordoma can or cannot be distinguished from peripheral chordoma. Both neoplasms share four cell types grouped in the form of nidi and cords: epithelioid cells, small glomoid cells, spindle cells, and cells with a vacuolated cytoplasm (physaliphorous cells) [5]. Although some authors claim that both conditions are in fact the same entity [6], the current state of the art is to regard them as two different neoplasms on the basis of their immunohistochemical and cytogenetic characteristics. Both tumors are positive for S-100 protein and the epithelial membrane antigen (EMA), but they differ in the expression of cytokeratins, with CK-10 and CK-19 eliciting a positive reaction in chordoma and a negative reaction in parachordoma [2, 7, 8]. In addition, only chordoma expresses the T-box transcription factor Brachyury, which participates in mesoderm differentiation and axial development [9].

Parachordoma has traditionally been considered a benign lesion whose treatment of choice is surgical resection. However, the recurrence rate has been found to be up to 20% by different studies, with follow-up periods ranging from three months to 12 years [4, 8, 10]. These relapses seem to result from the infiltration of adjacent soft tissues by small tumor nidi, which makes it essential to obtain tumor-free margins at the time of resection.

It should also be noted that seven of the cases published to date developed metastasis: in three cases the tumor invaded the lungs, in two it involved regional lymph nodes, and two patients had generalized metastasis. For that reason we considered it necessary to follow up these patients using the usual protocol for soft tissue sarcoma, subjecting them to periodic imaging studies.
